# Dr. Lewins on the Administration of Colchicum

**Published:** 1837-10

**Authors:** Robert Lewins

**Affiliations:** 6, Quality street, Leith


### DR. LEWINS ON THE ADMINISTRATION OF COLCHICUM.

*To the Editors of the British and Foreign Medical Review.*

Gentlemen: I have read with much satisfaction the notice you were pleased to take, in the July Number of the British and Foreign Medical Review, of a late paper of mine on Colchicum. Your remark is most just in regard to the tendency,—I had almost said exclusive direction—of medical energy, of late years, to pathological and physiological investigations, to mislead the attention of practitioners from the careful study and just appreciation of the effect of individual remedies on the animal economy in health and disease. Indeed, I have often thought that our most eminent pathologists sometimes appear much more desirous of having an opportunity of dissecting the bodies of the dead, than anxious to make any practical application of their post-mortem examinations for the benefit of the living! Permit me to say a few words in reply to your courteous and liberal criticism: in doing so, I am influenced solely by a desire to prosecute the investigation of the subject referred to, and not by any vain and idle wish to refute your opinion, or confirm my own where discrepancy exists between them.

Perhaps I expressed myself too strongly in regard to the mere *use* of colchicum by British practitioners; but I am still of opinion, from accurate information derived from private sources and from public medical charities, that it is much less extensively prescribed by a great proportion of the medical profession, than you, or that class of the profession with whom you are most familiar, imagine; and I adhere to my assertion that many, even of our best-informed physicians, are ignorant of the proper method of prescribing colchicum, as well as of the extent of its remedial efficacy. Need I produce other or more convincing proof of my averment than that contained in my original communication, where it is stated that a fatal dose had been lately administered by a learned physician in a public hospital? or that two drachms of the wine of the root, and half an ounce of a strong tincture of meadow-saffron seeds, are recommended as a proper dose by the late Dr. Duncan, unquestionably high authority on any subject connected with the materia medica.* But further, Gentlemen, on consulting Dr. Barlow's work, to which you had the goodness to refer me, to prove "that he at least is fully aware of the principles on which the potent drug should be prescribed," I was not a little surprised to find that the writings even of that deservedly eminent physician afford proof of the accuracy of my opinion on the disputed point. For, although Dr. Barlow undoubtedly entertains enlightened views on the therapeutical value of colchicum, as well as on every other medical subject, still the following directions as to the mode of prescribing it are, I fear, fraught with danger. "When colchicum is to be employed," says he, "it may be given either in full doses, so as to purge actively, or in divided doses frequently repeated. One drachm, one drachm and a half, or two drachms of the tincture of the seeds, should be administered at night, and repeated, if necessary, next morning. This quantity will generally purge briskly, but, if it fail, a third dose the following night will be pretty sure to succeed; at least, I have seldom found it necessary to exceed these doses." I should imagine not: indeed, from my experiments on the effects of colchicum on individuals in health and labouring under disease, I am convinced that such a dose of tincture of the seeds cannot be given with safety, unless the spirituous tincture of the seeds used by Dr. Barlow be a weaker preparation than that which I prescribe.†

In reference to your reasonable remarks as to my idea of the efficacy of colchicum in fever, it is true that the symptoms of none of my cases were such as to authorize me to report them as typhus; but I do think, from my knowledge of the history of that type of fever denominated typhus, as it occurs in this place, that some of the cases referred to would have degenerated into it, unless for the salutary effect produced by colchicum on the functions of organs vitally important in the animal economy.

Perhaps, like most other men who are convinced that the medicinal virtue of a remedy has been unknown or under-rated, I may err on the opposite side, and attach an importance to the one under consideration which more extensive experience will not confirm; but it is surely reasonable to expect very decided benefit, *in all kinds of fever*, from the active but judicious early administration of a medicine endowed with the power which colchicum unquestionably possesses on the nervous system in general, and on the gastric and hepatic systems in particular, as well as on the skin and bowels.

Other avocations have hitherto prevented me from continuing my contributions on the Physiological and Therapeutical Effects of Colchicum to our own metropolitan

* See Edinburgh New Dispensatory, 12th Edition, p. 953.

† The preparation of colchicum which I use is made by Messrs. Duncan and Flockhart, of whose pharmaceutical skill and accuracy it is impossible to speak in too high terms. The seeds, previously "passed through a mill," are infused for six weeks in Spanish white wine (sherry), in the proportion of two ounces of the seeds to sixteen ounces of wine.

Medical Journal; but I hope, by and by, to appear again there as a labourer in this field, when I shall consider myself honoured by the criticism of the Editors of the British and Foreign Medical Review, who always do their duty in a temper, tone, and spirit which show that they rightly understand the delicate nature of their important and difficult vocation.

I have the honour to be, Gentlemen, very respectfully yours,

ROBERT LEWINS.

6, *Quality street, Leith;* 31*st August*, 1837.

